# Supplementary figures and images for: Functional genomic mechanisms of opioid action and opioid use disorder: a systematic review of animal models and human studies
Source: Mol Psychiatry. 2023 Sep 15;28(11):4568–84. doi: 10.1038/s41380-023-02238-1 (PMC10914629; doi:10.1038/s41380-023-02238-1)

# Supplementary Figure 1

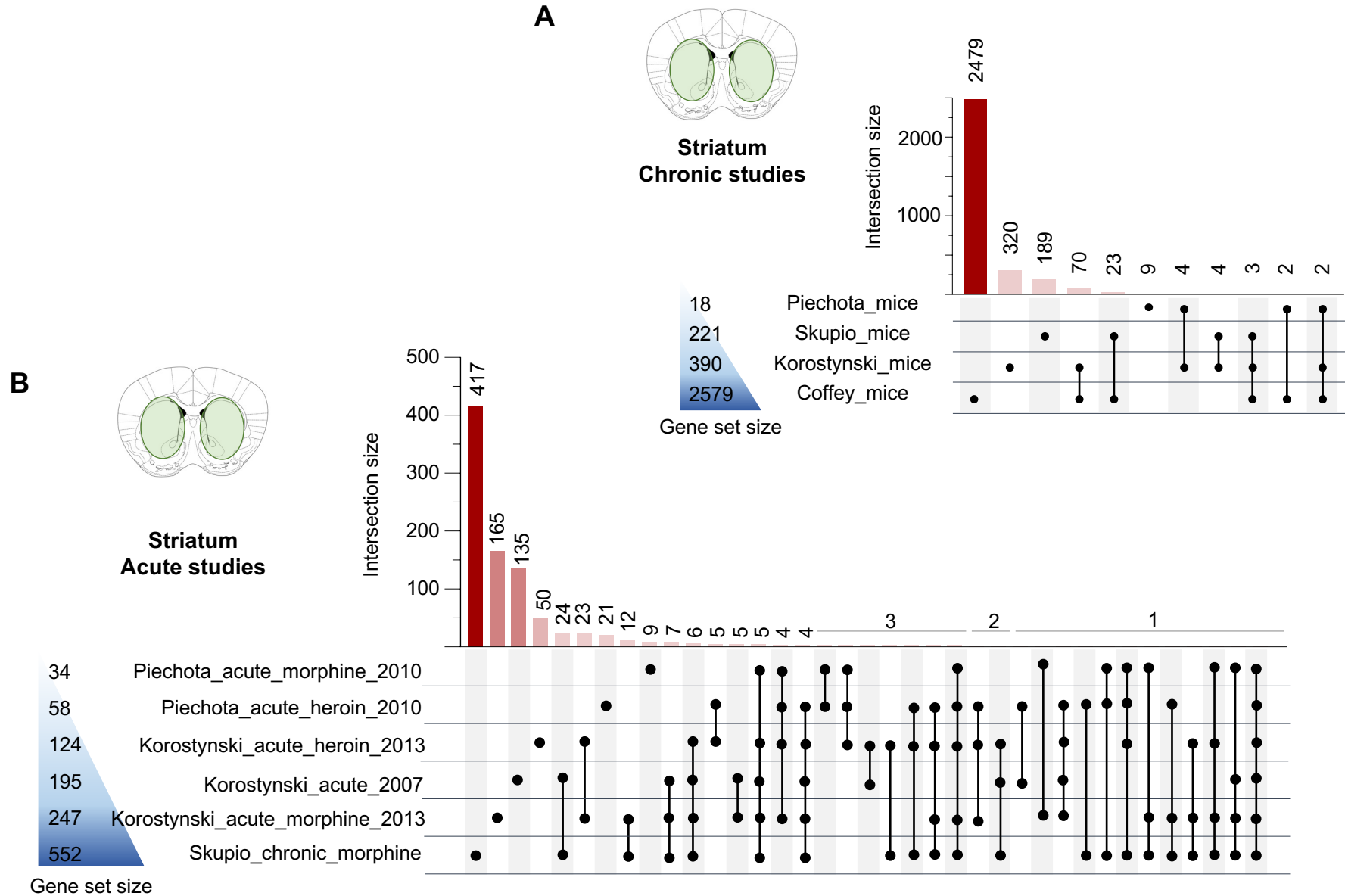

Supplement: Supplementary file 2 — Supplementary Figure 1 [file 41380_2023_2238_MOESM2_ESM.pdf]

Supplementary Figure 2

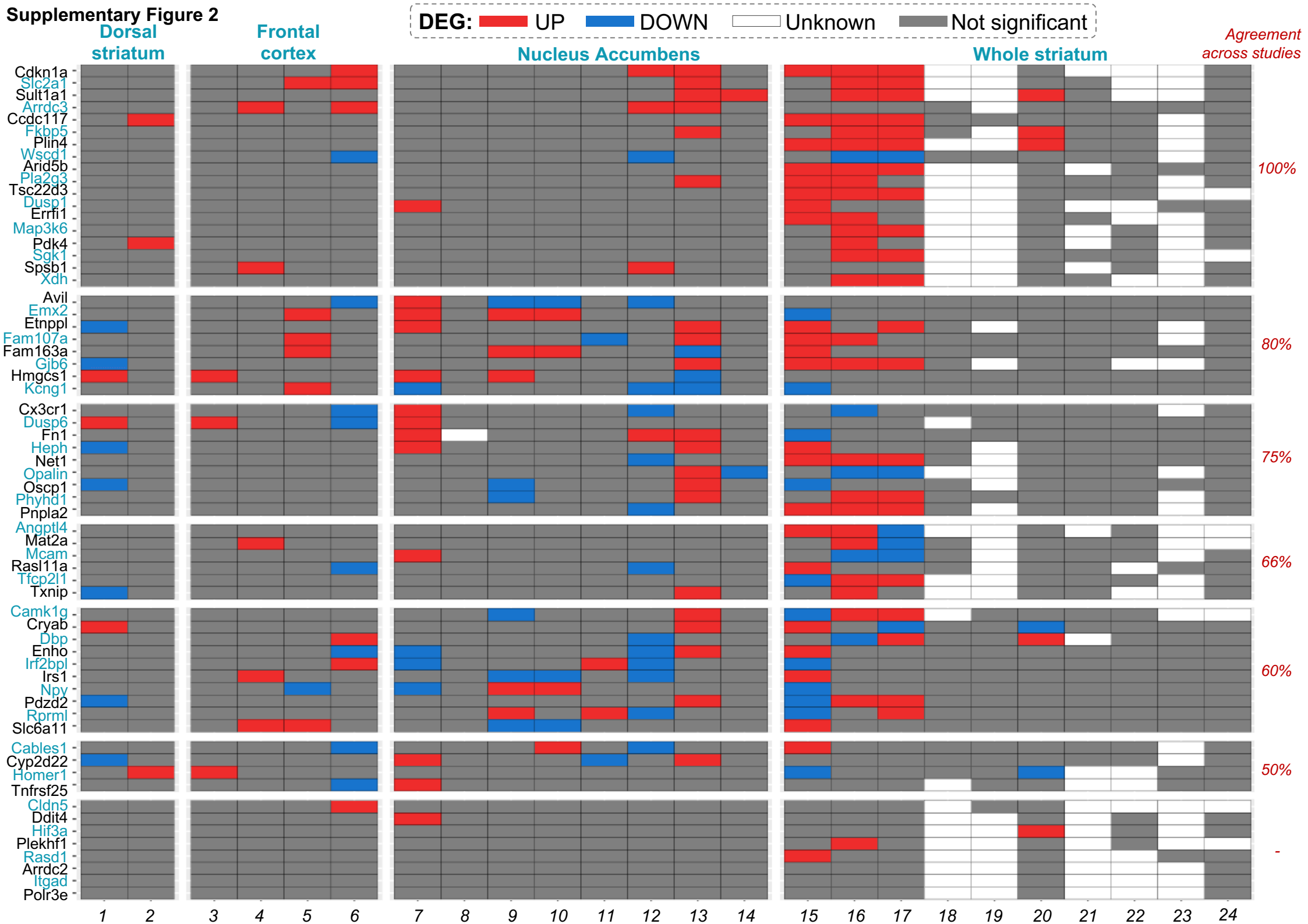

Supplement: Supplementary file 3 — Supplementary Figure 2 [file 41380_2023_2238_MOESM3_ESM.pdf]

Supplementary Figure 3

A

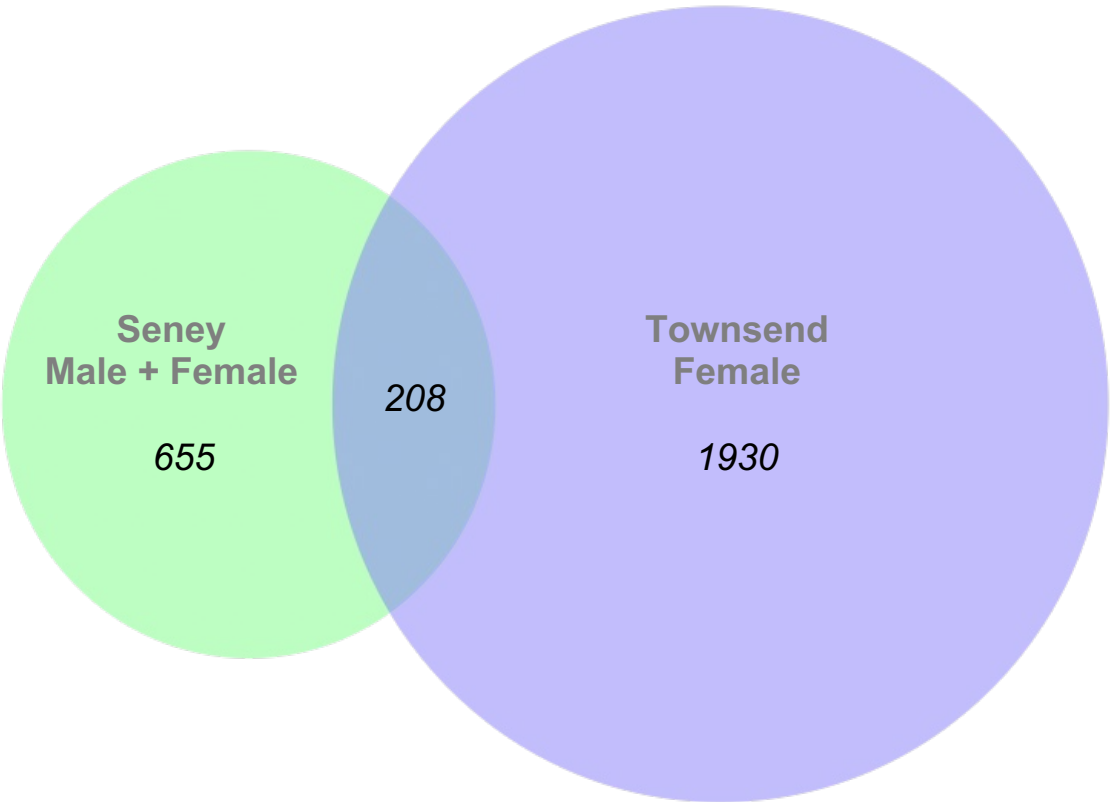

B

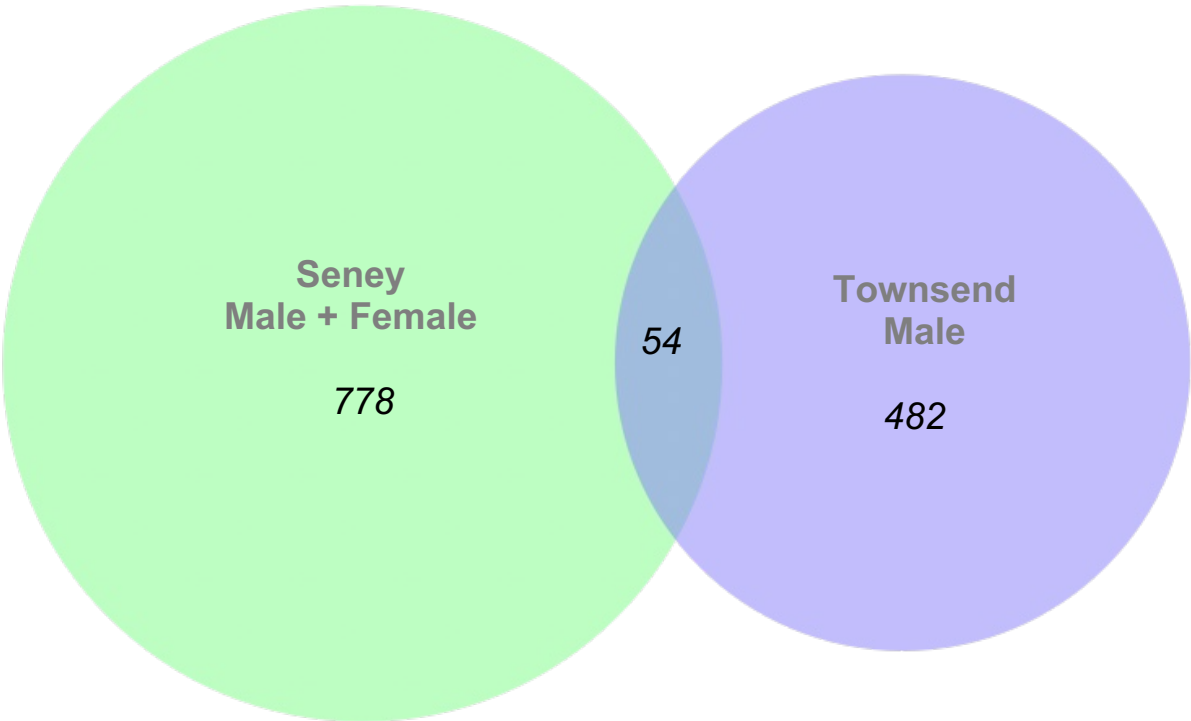

Supplement: Supplementary file 4 — Supplementary Figure 3 [file 41380_2023_2238_MOESM4_ESM.pdf]

Supplementary Figure 4

A

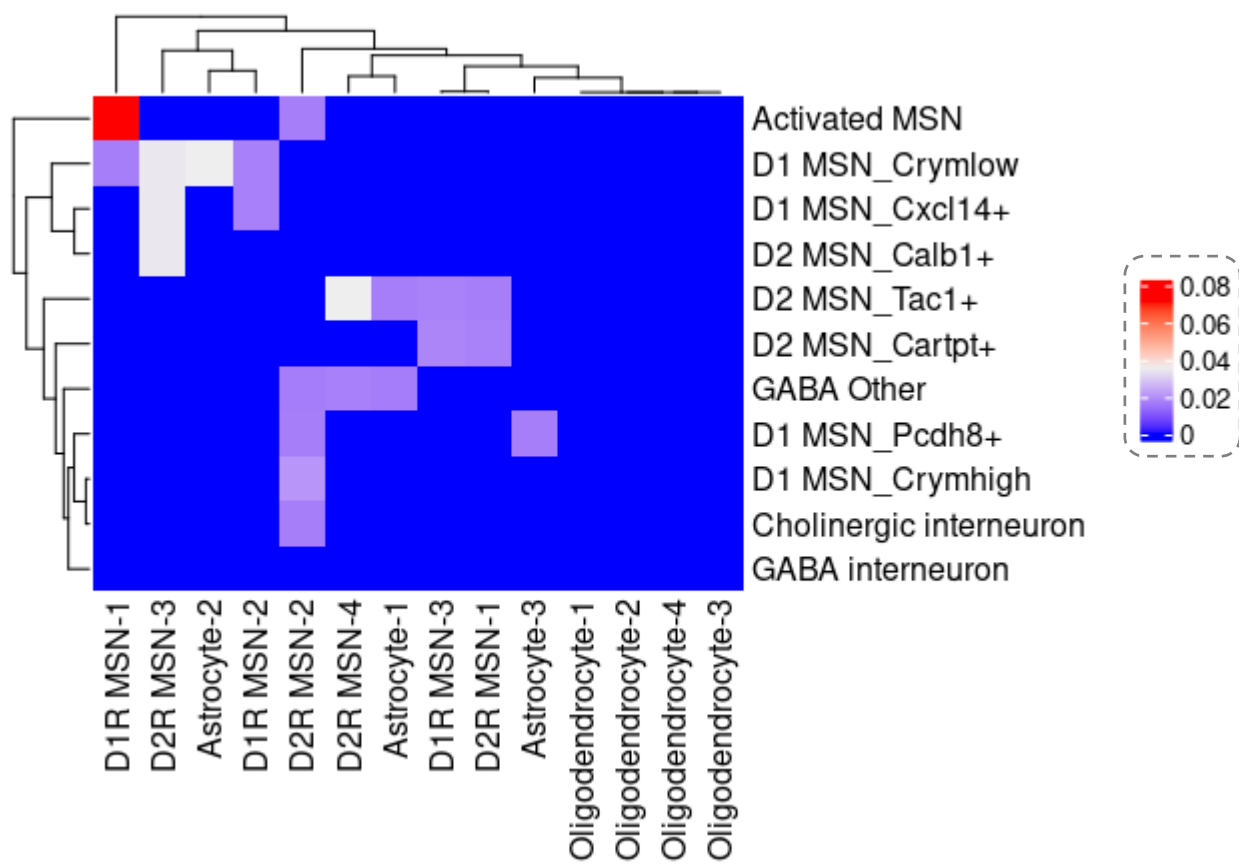

B

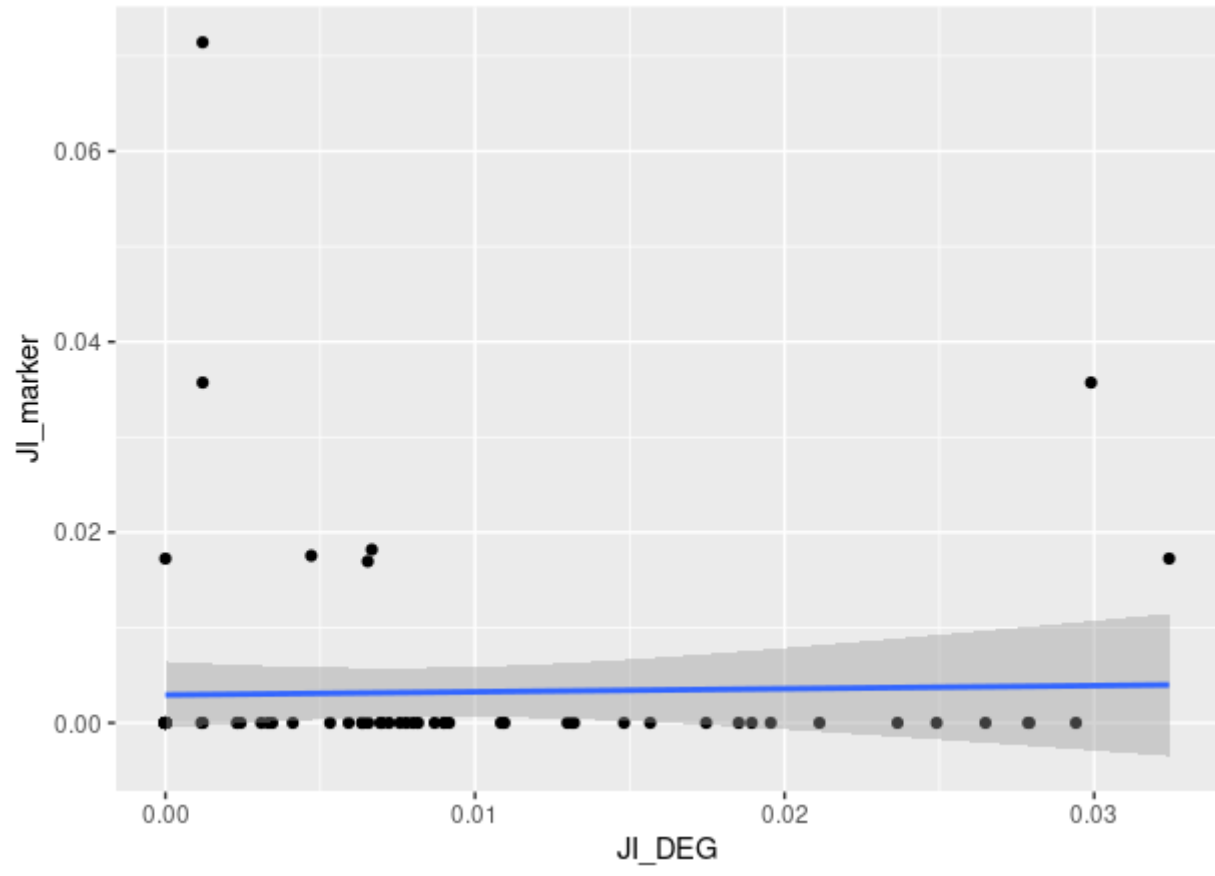

Supplement: Supplementary file 5 — Supplementary Figure 4 [file 41380_2023_2238_MOESM5_ESM.pdf]
